# Negative air ion exposure ameliorates depression-like behaviors induced by chronic mild stress in mice

**DOI:** 10.1007/s11356-022-20144-x

**Published:** 2022-04-11

**Authors:** Yun-Qing Hu, Ting-Ting Niu, Jian-ming Xu, Li Peng, Qing-Hua Sun, Ying Huang, Ji Zhou, Yu-Qiang Ding

**Affiliations:** 1 State Key Laboratory of Medical Neurobiology and MOE Frontiers Center for Brain Science, Institutes of Brain Science, Fudan University, No. 130 Dong’an Road, Shanghai, 200032 People’s Republic of China; 2 Institute of Occupational Health and Environmental Health, School of Public Health, Lanzhou University, No. 199 Donggang West Road, Lanzhou, Gansu 730000 People’s Republic of China; 3 Shanghai Typhoon Institute, CMA, No. 166 Puxi Road, Shanghai, 200030 People’s Republic of China; 4 Shanghai Key Laboratory of Meteorology and Health, Shanghai Meteorological Bureau, No. 166 Puxi Road, Shanghai, 200030 People’s Republic of China; 5 School of Public Health, Joint China-US Research Center for Environment and Pulmonary Diseases, Zhejiang Chinese Medical University, No. 548 Binwen Road, Hangzhou, 310053 People’s Republic of China; 6 Department of Laboratory Animal Science, Fudan University, No. 130 Dong’an Road, Shanghai, 200032 People’s Republic of China; 7 Department of Atmospheric and Oceanic Sciences & Institute of Atmospheric Sciences, Fudan University, No. 220 Handan Road, Shanghai, 200433 People’s Republic of China

**Keywords:** Negative ions, Air ions, Depression, CMS, Inflammation, Corticosterone

## Abstract

The presence of negative air ions (NAI) is suggested to be a beneficial factor in improving psychological status and used in treating depression as an alternative approach. However, more biological evidence from animal models is needed to ensure the effects of NAI on the mood regulation, through which can facilitate identification of possible underlying mechanisms. In this study, the chronic mild stress (CMS) protocol was used to induce depressive-like behaviors in mice, and the effects of NAI exposure on CMS-induced depression-like behaviors were examined. Thirty-day NAI exposure prevented the CMS-induced depression-like behaviors as shown by the restoration of sucrose preference and reduced immobility time in the tail suspension test. In addition, the elevation of serous corticosterone was present in CMS-treated mice but not existed in those with the NAI exposure. Furthermore, we observed altered ratios of some cytokines secreted by type 1 T helper (Th1) cells and Th2 cells in CMS-treated mice, but it could be restored after NAI exposure. In conclusion, NAI intervention is able to ameliorate CMS-induced depression-like behaviors in mice, and this effect is associated with the alteration of corticosterone and functional rebalance between Th1 and Th2 cells.

**Supplementary Information:**

The online version contains supplementary material available at 10.1007/s11356-022-20144-x.

## Introduction

Air ions are molecules of ionized particles present in the atmosphere and generated in a variety of natural or artificial ways (Jiang et al. 2018). It has been speculated that exposure to positive air ions is harmful to human health, while exposure to negative air ions (NAI) has beneficial health effects. Several explorations have focused on the biological effects of air ions on mood and behaviors (Bachman et al. 1966; Della Vecchia et al. 2020; Olivereau et al. 1981). A meta-analysis reported that the exposure to the air ions show no consistent results on the performances of wheel running, spontaneous locomotion, brain electrical activity, and sleep patterns in animals (Bailey et al. 2018), while other studies measuring the effects of air ionization on various psychological parameters related to mood or emotional state have demonstrated that higher concentrations of NAI exposure are positively associated with mental health (Chu et al. 2019; Jiang et al. 2018; Perez et al. 2013).

The etiology of depression is complex and diverse (Duman et al. 2016; Krishnan et al. 2008). Etiological hypotheses of this disorder include the dysfunctions of brain monoaminergic system, hyperactivity of hypothalamic–pituitary–adrenal (HPA) axis, inflammatory alterations, and neurotrophic abnormalities (Villas Boas et al. 2019). The hyperactivity of HPA axis is shown by the fact that high serous level of cortisol is present in a large population of depressed patients (Hinkelmann et al. 2009; Leonard 2018) and also in animal models (Wang et al. 2021; Zaletel et al. 2016). Cortisol (corticosterone in rodents) is released in response to stress and regulates immune and inflammatory processes, energy metabolism, and neuronal survival (Nikkheslat et al. 2018; Zunszain et al. 2011). Increasing data have evidenced that inflammation and HPA axis hyperactivity often coexist in the episodes of depression (Cernackova et al. 2020; Gold 2015).

Inflammation has been shown to interact with almost all pathophysiological domains known to be related to depression (Kim et al. 2016; Miller A. H. et al. 2009). Cytokines are polypeptides or glycoproteins synthesized and secreted by peripheral monocytes, macrophages, lymphocytes, and multiple cell types in the brain such as neurons, astrocytes, and microglia. They play important roles in the bidirectional immune communication between the brain and the periphery (Leonard 2018). Specifically, cytokines produced in the periphery can access and together with those generated within the brain influence the function state of the brain through humoral and neural pathways (Capuron et al. 2011). Cytokines affect many biological processes related to brain functions such as neurotransmitter metabolism and its activity, neuroendocrine, and neurocircuits relevant to mood, depression, and anxiety (Himmerich et al. 2019). Previous studies have found that the administration of interferon-γ (IFN-γ) and inflammation inducers, lipopolysaccharides, in rodents results in altered behaviors similar to those of depressed patients (Dantzer et al. 2008; Kentner et al. 2008). On the other hand, cytokine antagonists, such as interleukin-1 (IL-1) receptor antagonist and prebiotics, attenuate social and/or anxiety behavior in rodents (Arakawa et al. 2009; Dantzer 2004; Savignac et al. 2016). Besides, it has been reported that antidepressant treatment could decrease peripheral levels of IL-6, IL-10, tumor necrosis factor (TNF)-α, and IFN-γ (Chen et al. 2018; Kohler et al. 2018).

Chronic mild stress (CMS) is a crucial trigger of depression (Willner 2017), and CMS-treated animals exhibit depression-like behaviors, which can be reflected by increase of immobility time in the forced swimming and tail suspension tests and reduction of sucrose intake in the sucrose preference test (Chen et al. 2021; Garcia et al. 2009; Wang et al. 2021). The objective of this study is to investigate if NAI exposure affects depression-like behaviors in CMS-treated mouse model. To explore possible contribution of immune responses and HPA axis in this process, we measured contents of corticosterone and cytokines in the serum. Our results revealed that the NAI exposure could ameliorate CMS-induced depression-like behaviors in mice as shown by the data from the sucrose preference test and tail suspension test. In addition, the NAI exposure also interfered with the alterations of corticosterone and multiple cytokines in the serum of CMS-treated mice, which may contribute to the behavioral changes in CMS-treated mice.

## Material and methods

### Animals

Six-week-old male C57BL/6 mice were obtained from Shanghai Lingchang Biotechnology Co. Ltd, China. Animals were maintained in the specific pathogen-free animal facility and provided regular rodent chow and water ad libitum under a 12-h light/dark cycle (lights on at 7:00 a.m.), with a temperature of 25 ± 1 °C and a humidity level of 50 ± 10%. Prior to the experimental procedure, mice were accommodated in the experiment room for 2 weeks. All procedures were carried out in compliance with the Animal Experimental Ethics Committee (DSF-2020–041) of Shanghai Medical School, Fudan University.

### Apparatus

Filtered air (FA) box and NAI box (50 cm length × 40 cm width × 100 cm height) were equipped with a fan (ERF500D1N, Honeywell). The NAI box contained a negative ion generator (Shanghai Sailumei Environmental Protection Technology Co., Ltd, China) and provided approximately 4 × 10^4^ small NAIs per cubic centimeter (high-density exposure) at 1 m to the feeding cages; this concentration was determined by a combination of the clinical data and previous studies in mouse (Bailey et al. 2018; Flory et al. 2010; Terman et al. 1995). Automatic observation system of atmospheric negative ions (Wide Creative Science & Technology, Beijing, China) was used to monitor the concentration of NAIs whose ion mobility is not less than 0.4 cm^2^/(V·s).

### Experimental procedure

Mice were housed either in the FA box or NAI box. In each box, animals were randomly assigned into two groups: one suffered from CMS for 30 days and the other served as control without CMS treatment. Therefore, the research was consisted of 4 groups: (1) control mice in FA box, (2) CMS-treated mice in FA box, (3) control mice in NAI box, and (4) CMS-treated mice in NAI box. Mouse body weight between groups was evaluated before and after 30-day exposure. After the 30-day exposure, behavioral tests were performed, and mice were sacrificed after completing these tests for examination of contents of corticosterone and cytokines in the serum. Blood samples for corticosterone assays were collected at 2:00 p.m.

### CMS paradigms

The CMS protocol was conducted according to the well-established methods with minor modifications (Chen et al. 2021; Wang et al. 2021; Zhao et al. 2021). The mouse was restrained in a 50-mL tube with no space to turn over for 6 h every day, along with unpredictable 3-min shaking for 5–7 times during this period.

### Behavioral testing

Behavioral experiments were performed in a sound-proof room with a neutral environment. All behavioral tests were conducted during the light phase of the light/dark cycle. All mice were given a 30-min habituation in the behavioral room before the test. After each animal completed the behavioral test, the equipment was thoroughly cleaned to eliminate olfactory effects. The experimenter was blind to the group identity of the tested mice. Some behavioral tests were recorded by a video camera, and then the footages were analyzed by a trained researcher.

#### Open field test

A black square arena (45 × 45 × 30 cm) was used to examine locomotor activity. Mice were placed in the center of the arena and allowed to explore the apparatus freely for 5 min (Wang et al. 2018). Total distance moving in the field was analyzed by the EthoVision XT video tracking software (Ver. 12).

#### Sucrose preference test

All stages of the test were carried out at the same time of the day (Zhang et al. 2019). Each cage was provided with two drinking tubes containing sucrose water (2% w/v) during the first 24-h training phase. Then the next day, one bottle with 2% sugar solution and another bottle with regular water were provided to mice. After training, mice were deprived of water and food for 24 h, then the mice were given the free choice to drink from two bottles for 24 h: one was filled with a sucrose solution, and the other was filled with water. The positions of the bottles in the cage were switched after the first 12 h. Sucrose and water consumptions were recorded separately before and after the test. Sucrose preference % = (sucrose intake/total intake) × 100%; lower preference serves an indicator of increased depressed-like behaviors. The total intake value is the sum of water intake value and sucrose intake value.

#### Forced swimming test

Animals were individually placed in a transparent acrylic cylinder (height 30 cm, diameter 15 cm) for 6 min (Zhao et al. 2021), which was filled with tap water to a depth of 20 cm. The first 2 min were spent for adaptation, and the last 4 min were analyzed. Immobility time was evaluated as floating or no active movements except those necessary for the mouse to keep its head above water. Longer immobility time serves as an indicator of increased depression-like status.

#### Tail suspension test

In the TST, mice were suspended 30 cm above the floor with an adhesive tape applied approximately 1 cm from the end of the tail on a metal hook (Xu et al. 2021). At the beginning of the test, nearly all the mice attempted to escape from hanging, but after a period of struggling, it showed intermittent immobility, displaying a state of “behavioral despair.” The duration of this state was considered as the immobility time. The activities of the mice were recorded by a video camera, and then the immobility time during the last 4 min of a 6-min testing period was evaluated. Again, longer immobility time serves as an indicator of increased depression-like status.

#### Rotarod test

Mice were habituated to the rod for 2 min while it slowly rotated (10 rpm-rotations per minute), and they were replaced on the rod if they fell off during the 2 min. Testing consisted of three trial sessions; each session had a progressively increasing speed from 4 to 40 rpm within 5 min. On each of the three trials, the mouse was placed on the rod and left there until either 300 s elapsed or until the mouse fell off. There was a 1-h break between trials. The average performance of total time on the rod for the three-trial session was analyzed.

#### Y maze

Working memory was evaluated by spontaneous alternation Y-maze test (Pontifex et al. 2021). The apparatus comprised three identical arms (30 cm × 5 cm × 10 cm), spaced 120° apart. The mouse was placed in one arm of the maze and allowed to explore freely for 8 min. At the same time, zone transitions were recorded by tracking software (EthoVision XT video tracking software, Ver. 12). Spontaneous alternation was calculated using the following formula: spontaneous alternation % = (number of alternations/total arm entries − 2) × 100.

### Cytokine and corticosterone measurement

Blood was collected from orbital sinus in anesthetized mice. The serum from blood was isolated by centrifugation for 20 min at 1000 × g at 4 ℃; then, the aliquots of the samples were stored at – 80 °C before they were measured. Cytokines, including granulocyte-colony stimulating factor (G-CSF), granulocyte–macrophage colony-stimulating factor (GM-CSF), IL-1α, IL-1β, IL-2, IL-3, IL-4, IL-5, IL-6, IL-7, IL-9, IL-10, IL-12p70, IL-13, IL-15, IL-17, IL-21, IL-23, IFN-γ, and TNF-α in the serum, were determined using commercial Interleukin Antibody Arrays (QAM-INT-1, RayBiotech Inc.). Corticosterone content was determined by using an enzyme-linked immunosorbent assay kit (Enzo, ADI-900–071). Experiments were performed in accordance with the manufacturer’s protocols. The concentrations of IL-4, 5, 6, 10, and 13 were divided by those of IL-2 and IFN-γ, respectively, and the ratios were used to evaluate functional balance of anti-inflammatory and proinflammatory cytokines.

### Statistical analysis

All figures were performed using GraphPad Prism 6 and IBM SPSS statistics 20.0. Data were expressed as the mean ± standard error of mean (SEM). One-way analysis of variance (ANOVA) was used for statistical analysis of data, followed by post hoc multiple comparisons with Bonferroni (test of homogeneity of variance α is more than 0.05) or Tamhane’s T2 (test of homogeneity of variance α is less than 0.05) correction. Statistical significance was defined as *P < 0.05, **P < 0.01, ***P < 0.001.

## Results

### NAI exposure ameliorates CMS-induced depression-like behaviors

**Fig. 1:**
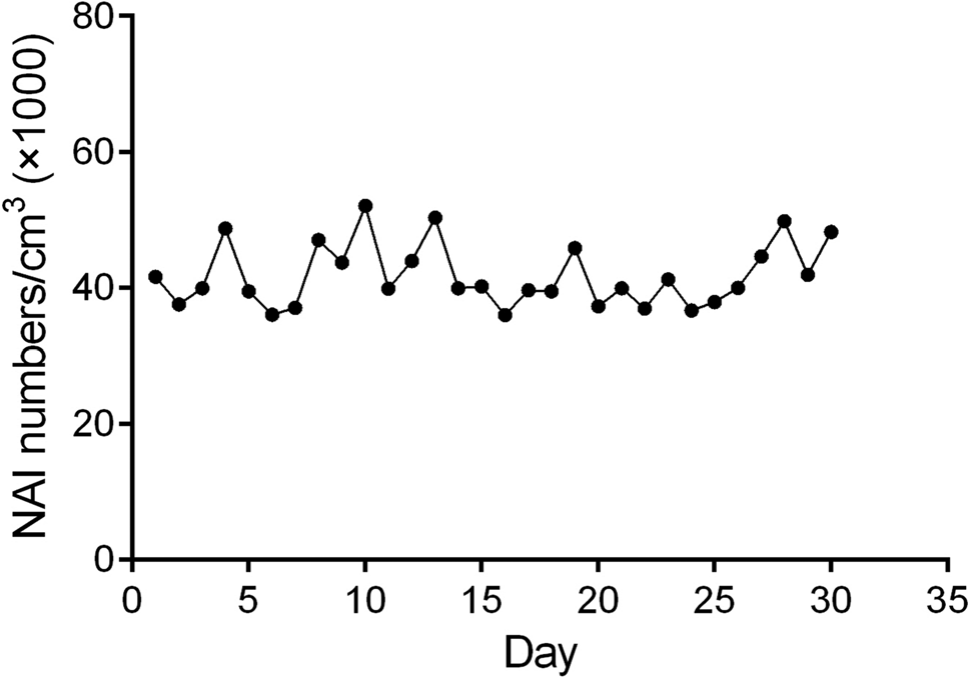
The concentration of negative air ions detected daily. Six air samples per minute to detect the concentration, removes the maximum and minimum values, and average value of the remaining 4 times are shown by NAI numbers/cm^3^ in Y axis

During the 30-day experiment, the concentration of negative oxygen ions was monitored (Fig. 1), showing a consistent supply of NAIs.

**Fig. 2:**
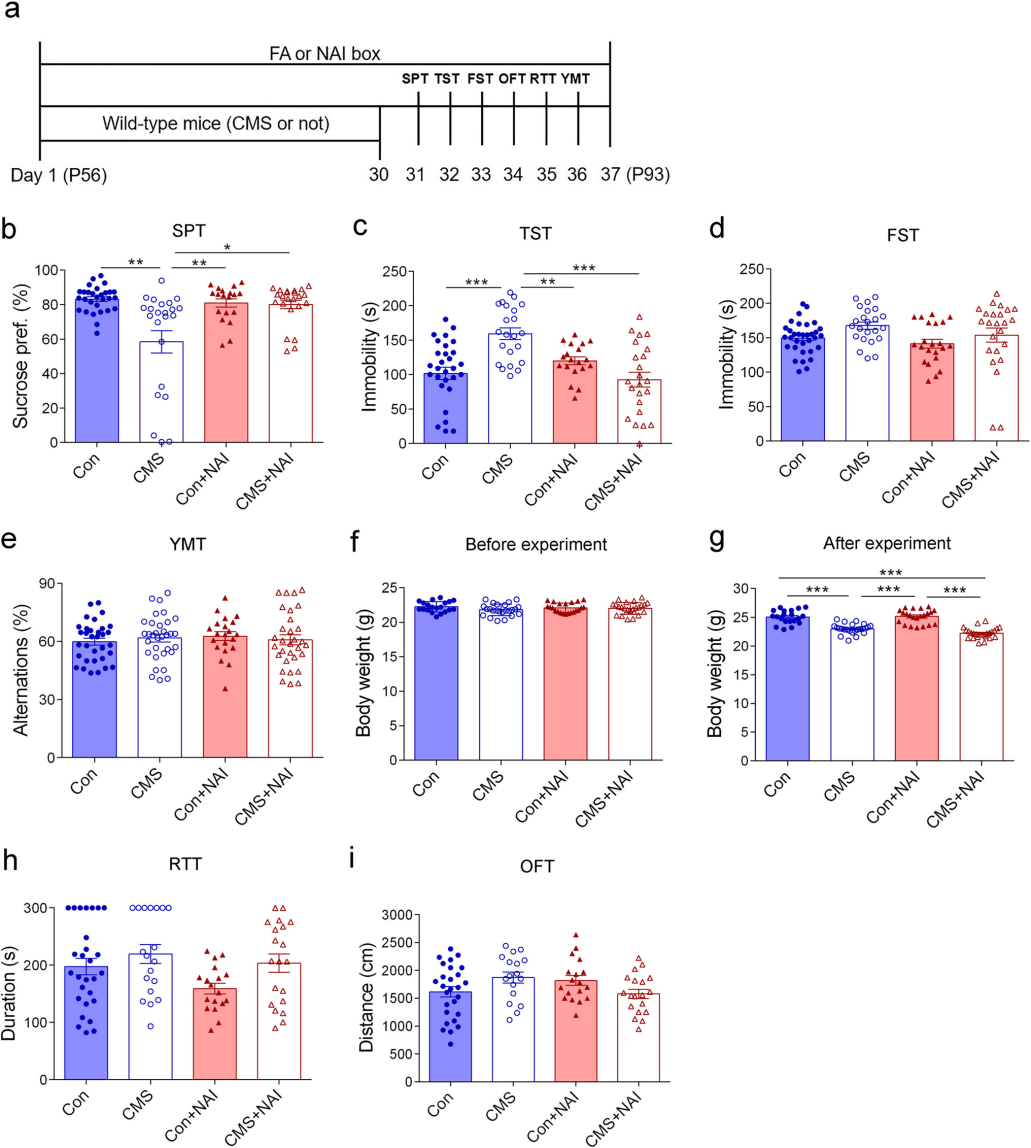
NAI intervention alleviates depression-like behavior in mice after stress exposure. **a** Diagram of experiment design and timeline. **b** Sucrose preference in the Sucrose preference (SPT). The one-way ANOVA test measured the significant differences between these groups {*F*[3,92] = 11.247, ***P < 0.0001; Tamhane’s T2 multiple comparisons test showed **P = 0.003 (Con vs. CMS); *P = 0.012 (CMS vs. CMS + NAI); **P = 0.009 (CMS vs. Con + NAI)}. **c** Time spent immobile in the tail suspension test (TST). The one-way ANOVA test measured the significant differences between these groups {*F*[3,90] = 10.431, ***P < 0.0001; Tamhane’s T2 multiple comparisons test showed ***P < 0.0001 (Con vs. CMS); **P = 0.003 (CMS vs. Con + NAI); ***P < 0.0001 (CMS vs. CMS + NAI)}. **d** Time spent immobile in the forced swimming test (FST). The one-way ANOVA test with Tamhane’s T2 multiple comparisons test measured the significant differences between these groups {*F*[3,98] = 2.475, *P* = 0.066}. **e** Spontaneous alternation in Y maze test (YMT). The one-way ANOVA test with Bonferroni multiple comparisons test measured the significant differences between these groups {*F*[3,122] = 0.366, *P* = 0.778}. **f** Body weight evaluated before the experiment. The one-way ANOVA test with Bonferroni multiple comparisons test measured the significant differences between these groups {*F*[3,87] = 2.509, *P* = 0.064}. **g** Body weight evaluated after 30-day CMS treatment in mice with or without NAI exposure. The one-way ANOVA test measured the significant differences between these groups {*F*[3,87] = 45.286, ***P < 0.0001; Bonferroni multiple comparisons test showed ***P < 0.0001 (Con vs. CMS); ***P < 0.0001 (Con vs. CMS + NAI); ***P < 0.0001 (CMS vs. Con + NAI); ***P < 0.0001 (CMS + NAI vs. Con + NAI)}. **h** Time spent on the rod in the rotarod test (RTT). The one-way ANOVA test with Tamhane’s T2 multiple comparisons test measured the significant differences between these groups {F[3,81] = 2.681, *P* = 0.052}. **i** Distance traveled in the open field test (OFT). The one-way ANOVA test with Bonferroni multiple comparisons test measured the significant differences between these groups {*F*[3,77] = 2.338, *P* = 0.08}. *P < 0.05, **P < 0.01, ***P < 0.001. Data are presented as mean ± S.E.M

The CMS design and timeline of behavioral observations are shown in Fig. 2a. The reduction of preference to sucrose reflects the core depression-like behaviors in CMS-induced depression animal model (Czeh et al. 2016). As expected, the CMS treatment reduced the consumption of sucrose solution in FA group (Fig. 2b). Importantly, the negative ion intervention restored the sucrose preference in CMS-treated mice in NAI group compared with those in FA group (Fig. 2b).

To further evaluate the effect of NAI on depression-like behaviors, the tail suspension test and forced swimming tests were performed. In the tail suspension test, the CMS treatment led to significantly increased immobility time in FA group, but did not do so in NAI group as shown by similar immobility time compared with control mice in NAI group (Fig. 2c). However, in the forced swimming test, there were no significant differences between control and CMS-treated mice in either FA or NAI group (Fig. 2d). In addition, short-term memory (Czaczkes 2018), a type of working memory responsible for the temporary storage of information, was examined using Y maze, and there were no differences among the four groups (Fig. 2e).

It is well known that the CMS treatment prevents the increase of body weight during the 1-month CMS period (Lu et al. 2014). The body weight before the experiment was not significantly different among the four groups (Fig. 2f). After 30-day experiment, in FA group, there was no weight gain in the CMS-treated mice, whereas control mice gained more weight than before (Fig. 2f, g). However, the unchanged body weight during the research in CMS-treated mice was also present in NAI group (Fig. 2f, g; Fig. S1), showing that the NAI exposure has no effects on the alteration of body weight in CMS-treated mice, although it ameliorates CMS-induced depression-like behaviors as mentioned above.

To exclude the possibility that the behavioral alterations were associated with inability of locomotion activity, we carried out the rotarod test and open-field test, which are used to examine motor coordination and spontaneous locomotion (Moniruzzaman et al. 2018; Ramshini et al. 2018). It was found that there was no significant difference among the groups in the two tests in terms of time stayed on the rod and traveled distance in the open field (Fig. 2h, i). Taken together, we demonstrate that NAI exposure prevents the occurrence of “anhedonia” behavior and some aspects of “despair” behaviors induced by the CMS treatment.

### Effects of NAI exposure on corticosterone levels of CMS-treated mice

**Fig. 3:**
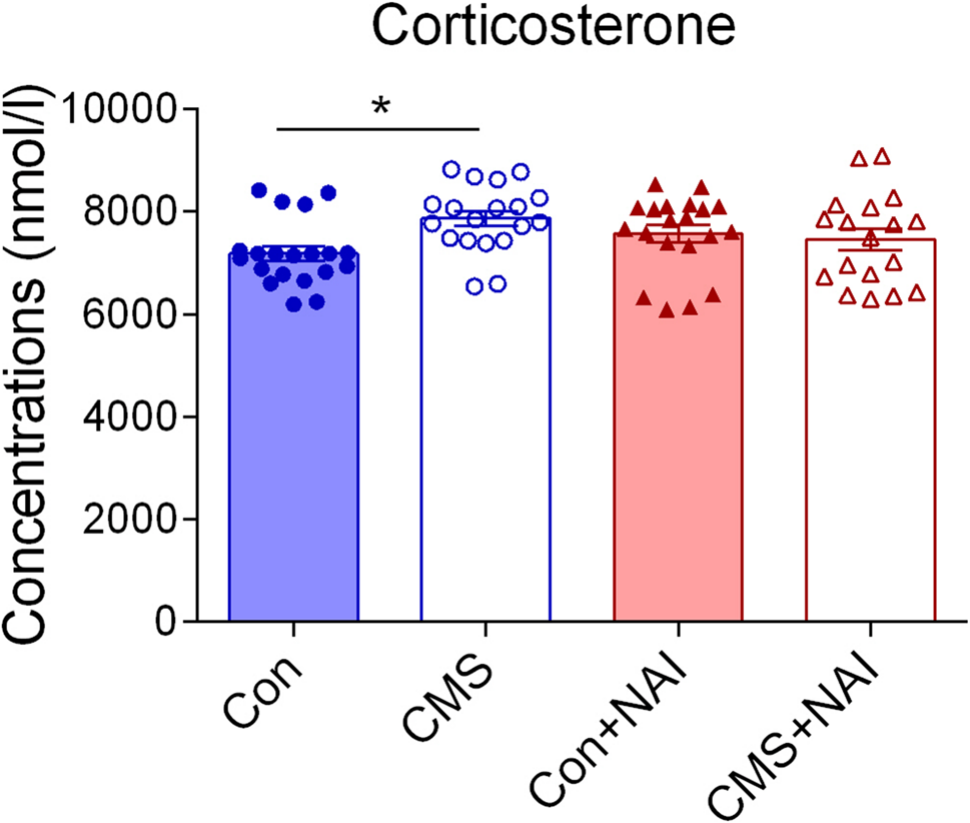
Effects of NAI intervention on corticosterone levels in the serum of mice with CMS. A significant difference is observed between control (Con) and CMS-treated (CMS) mice in FA group but not NAI group. The one-way ANOVA test measured the significant differences between these groups {*F*[3,73] = 2.924, *P = 0.039; Bonferroni multiple comparisons test showed *P = 0.027 (Con vs. CMS)}

Hyperactivity of the HPA axis is one of well-documented factors in the etiology of depression, which can be reflected by the increased level of cortisol in serum (Dean et al. 2017; Kim et al. 2016). Consistently, the CMS treatment did induce an increase of the concentrations of serous corticosterone in FA group, but not in CMS-treated mice in NAI group (Fig. 3), showing that 1-month NAI exposure relieves the HPA axis hyperactivity induced by CMS in mice.

### Effects of NAI exposure on cytokine levels of CMS-treated mice

**Fig. 4:**
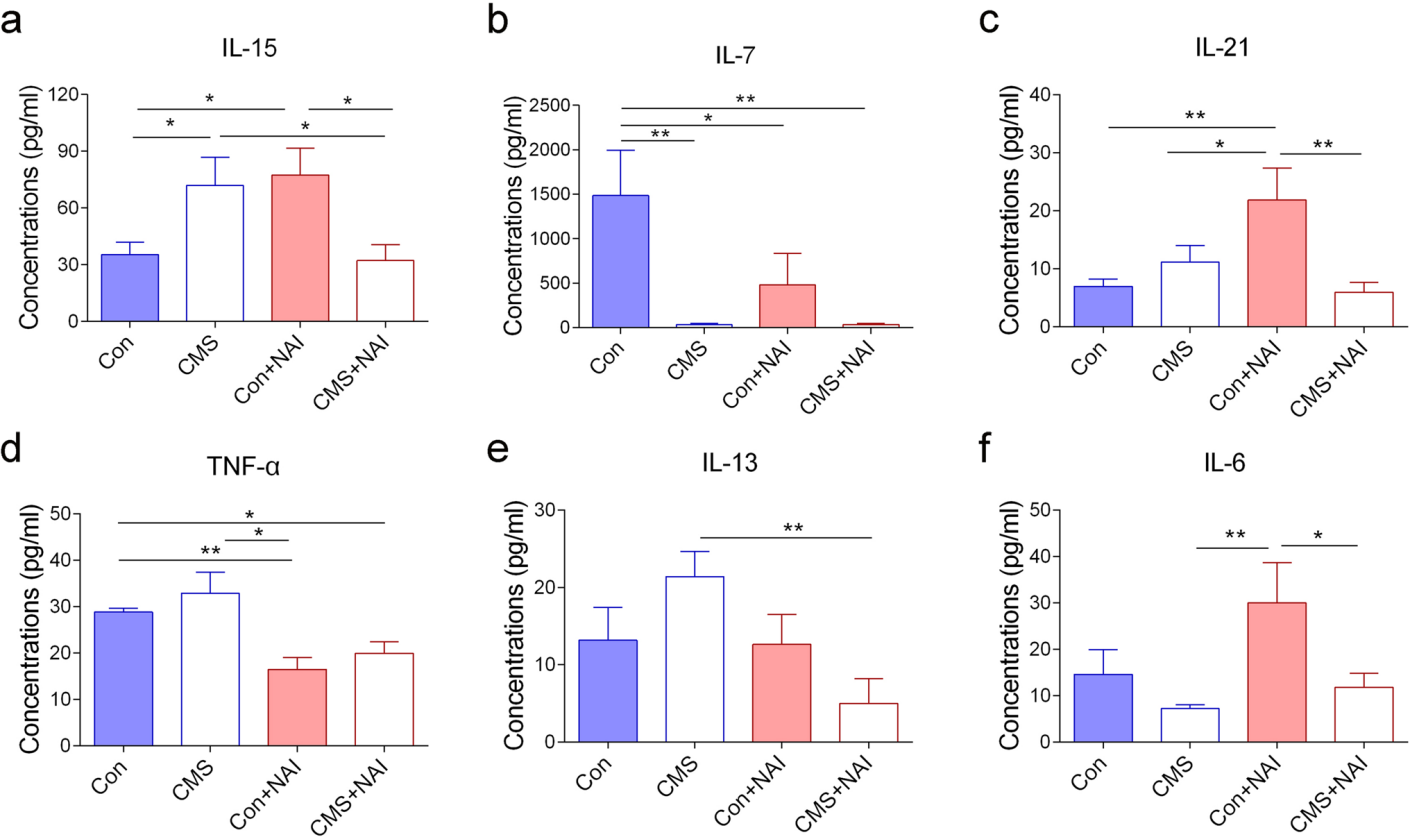
Effects of NAI intervention on inflammatory cytokine levels in serum of mice with CMS. **a** The concentration of IL-15. The one-way ANOVA test measured the significant differences between these groups {*F*[3,35] = 4.055, *P = 0.014; Bonferroni multiple comparisons test showed *P = 0.033 (CMS vs. Con); *P = 0.015 (Con + NAI vs. Con); *P = 0.024 (CMS vs. CMS + NAI); *P = 0.011 (CMS + NAI vs. Con + NAI)}. **b** The concentration of IL-7. The one-way ANOVA test measured the significant differences between these groups {*F*[3,34] = 4.485, **P = 0.009; Bonferroni multiple comparisons test showed *P* = **0.003 (CMS vs. Con); **P = 0.003 (CMS + NAI vs. Con); *P = 0.032 (Con vs. Con + NAI)}. **c** The concentration of IL-21. The one-way ANOVA test measured the significant differences between these groups {*F*[3,35] = 4.703, *P* = **0.007; Bonferroni multiple comparisons test showed **P = 0.004 (Con vs. Con + NAI); *P = 0.029 (CMS vs. Con + NAI); **P = 0.002 (CMS + NAI vs. Con + NAI)}. **d** The concentration of TNF-α. The one-way ANOVA test measured the significant differences between these groups {*F*[3,35] = 6.485, **P = 0.001; Tamhane’s T2 multiple comparisons test showed *P = 0.033 (CMS + NAI vs. Con); **P = 0.005 (Con vs. Con + NAI); *P = 0.044 (CMS vs. Con + NAI)}. **e** The concentration of IL-13. The one-way ANOVA test measured the significant differences between these groups {*F*[3,34] = 3.328, *P = 0.031; Bonferroni multiple comparisons test showed **P = 0.003 (CMS vs. CMS + NAI)}. **f** The concentration of IL-6. The one-way ANOVA test measured the significant differences between these groups {*F*[3,33] = 3.304, *P = 0.032; Bonferroni multiple comparisons test showed **P = 0.006 (CMS vs. Con + NAI); *P = 0.025 (CMS + NAI vs. Con + NAI)}. (*P < 0.05, **P < 0.01). 9 ≤ *n* ≤ 11/group

The CMS treatment increases the levels of corticosterone, which is known to negatively regulate immune responses (Villas Boas et al. 2019; Zaletel et al. 2016). To explore possible mechanisms underlying the effects of NAI exposure on CMS-induced depression-like behaviors, we next measured the contents of a panel of cytokines in the serum, based on the concept that cytokines play important roles in immune responses and HPA axis activation (Kim et al. 2016). Comparing the data from control and CMS-treated mice in FA group would be useful to evaluate if CMS treatment itself interferes the serous levels of cytokines. Our results showed that the levels of IL-15 were upregulated in CMS-treated mice, while those of IL-7 were downregulated in the serum (Fig. 4a, b). Comparing the data from control mice in NAI and FA groups would provide the information if the NAI exposure itself has any contribution to alterations of cytokines, and it showed that the levels of IL-15 and IL-21 were increased, and those of IL-7 and TNF-α were decreased (Fig. 4a–d). In this study, a total of 20 cytokines were examined, and only a small number of them displayed the changes in the serum, showing that specific cytokines are affected by the CMS or NAI exposure. The data showing unchanged levels of cytokines are included in Fig. S2.

Next, we asked if the NAI exposure contributes to the alteration of cytokines in CMS-treated mice. The increased levels of IL-15 were no longer existed, while the deceased levels of IL-7 were still present in CMS-treated mice with the NAI exposure, as compared with those without NAI exposure (Fig. 4a, b). There were increasing tendency of IL-13 and TNF-α levels in CMS-treated mice although the *p* value was not statistically significant relative to control mice in FA group; while in NAI group, their levels of CMS-treated mice were reduced to the control levels (Fig. 4d, e). These results showed that the NAI exposure interferes with the changes of serous cytokines in CMS-treated mice.

**Fig. 5:**
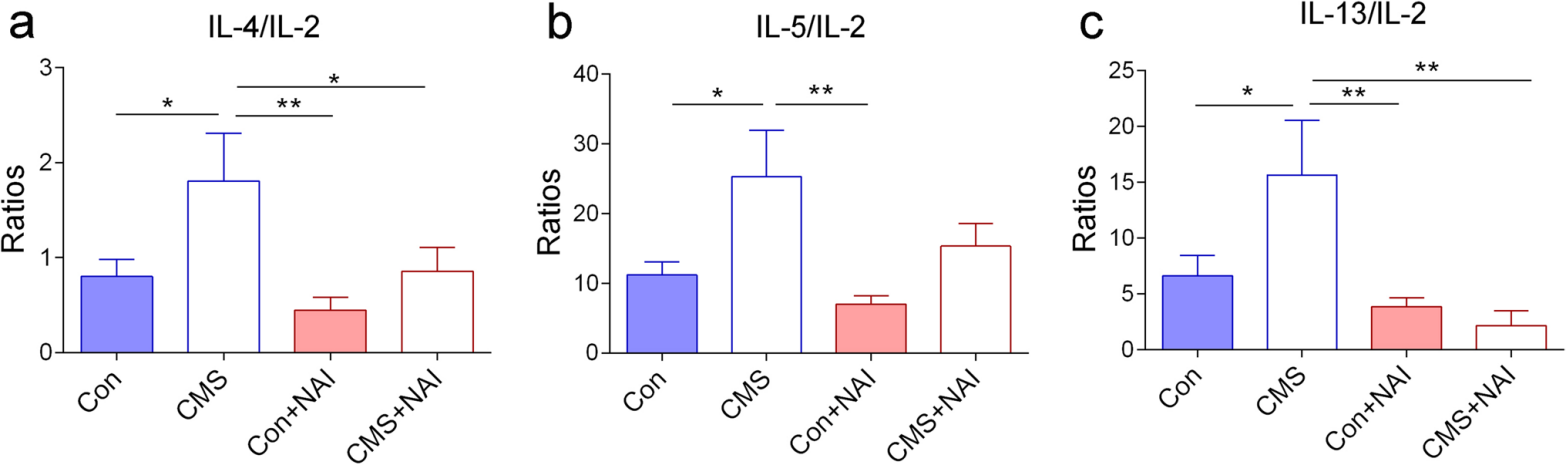
Comparison of ratios of IL-4/IL-2, IL-5/IL-2, and IL-13/IL-2 in CMS-treated mice with and without NAI exposure. **a** The ratio of serous concentration of IL-4/IL-2. The one-way ANOVA test measured the significant differences between these groups {*F*[3,35] = 3.599, *P = 0.023; Bonferroni multiple comparisons test showed *P = 0.030 (CMS vs. Con); *P = 0.035 (CMS vs. CMS + NAI); **P = 0.003 (CMS vs. Con + NAI)}. **b** The ratio of serous concentration of IL-5/IL-2. The one-way ANOVA test measured the significant differences between these groups {*F*[3,35] = 3.981, *P = 0.015; Bonferroni multiple comparisons test showed *P = 0.018 (CMS vs. Con); *P* = **0.002 (Con + NAI vs. CMS)}. **c** The ratio of serous concentration of IL-13/IL-2. The one-way ANOVA test measured the significant differences between these groups {*F*[3,33] = 5.016, *P* = 0.006; Bonferroni multiple comparisons test showed *P = 0.024 (CMS vs. Con); **P = 0.001 (CMS + NAI vs. CMS); **P = 0.003 (CMS vs. Con + NAI)}. (*P < 0.05, ^*^*P < 0.01). 9 ≤ *n* ≤ 11/group

Leukocytes, especially T cell population, play a key role in immune responses. Th1 cells and Th2 cells are the two major subgroups of T cells that are characterized primarily on the basis of cytokines secreted (Varade et al. 2021). Th1 cells secret type I cytokines (e.g., IL-2 and IFN-γ) which are mainly pro-inflammatory, while Th2 cells secrete type II cytokines (e.g., IL-4, IL-5, IL-6, IL-10, and IL-13) which are mainly anti-inflammatory (Gharagozloo et al. 2013; Maher et al. 2014). Accumulated evidence supports the idea that stress leads to an increase of pro-inflammatory cytokines (Li et al. 2016, 2020). However, the data from animal models and clinical research revealed significant variabilities in type I and type II cytokine profiles (Cuervo et al. 2021; Koivisto et al. 2019; Razali et al. 2020). It seems to be clear that the examination of a single cytokine or small groups of cytokines is not sufficient to evaluate the alterations of cytokines in depression-related animal models. Instead, one way to gain a better insight might be achieved by examining the ratios between the two types of cytokines, which reflects the balance between them and the tilt of immune response (Rostaing et al. 1999; Yoon et al. 2012). To this end, the ratios of type II (i.e., IL-4, IL-5, IL-6, IL-10, and IL-13) and type I cytokines (i.e., IL-2 and IFN-γ) were calculated. We found that these ratios of IL-4/IL-2, IL-5/IL-2, and IL-13/IL-2 were elevated in CMS-treated mice, and remarkably, the elevations of IL-4/IL-2 and IL-13/IL-2 were prevented by NAI exposure (Fig. 5a, c). There is also a decreased tendency of the ratio of IL-5/IL-2 after NAI exposure, although the *p* value was not statistically significant (Fig. 5b). The others were not significantly different, and the data are shown in Fig. S3. Taken together, it can be concluded that the NAI exposure is likely having the ability of preventing the functional shift of cytokines to inflammatory status in CMS-treated mice.

## Discussion

In this study, we showed that NAI intervention can improve the depression-like behaviors and prevent the increase of serous corticosterone in CMS-treated mice. The data from the comparison of cytokine ratios suggest a role of NAI intervention in functional rebalancing type I and type II cytokines in CMS-treated mice.

The core depressive symptom of CMS model is decreased sucrose preference in rodents (Czeh et al. 2016; Willner 2005), and it was confirmed in our CMS-treated mice. The role of NAI exposure in alleviating depression-like behaviors is supported by the data of the restoration of sucrose preference and the reduction of immobility time in the tail suspension test in CMS-treated mice. However, the immobility time in the forced swimming test was not different between the CMS-treated mice with and without NAI exposure, showing that our CMS protocol does not fully reflect depression-like behaviors and thus is not an ideal model for evaluation of the effects of NAI intervention on depression-like behaviors under our experimental condition.

Clinical data have shown that high-density negative air ionization is effective in treating seasonal affective disorders including major depression (Terman et al. 2006; Terman et al. 1998). In addition to therapeutic application, it is also recommended that NAI exposure either in natural environment or by artificial way can be used in preventing the onset of mental illness for people who had experienced chronic/acute stress and/or displayed a trend of developing the disorders. Thus, the previous and present findings strongly support the idea that NAI intervention is a useful tool or providing as an alternative way in treating depression.

Glucocorticoids are the final products of the HPA axis and regulate stress-triggered responses through a negative feedback with the hypothalamus and pituitary gland (van Bodegom et al. 2017). A wealth of evidence documented that chronic stress exposure leads to an impairment of the negative feedback of the HPA axis with increased levels of cortisol (Barfield et al. 2018; Kvarta et al. 2015). The hyperactivity of HPA axis is observed in the majority of depressed patients (Nemeroff et al. 2005; Pruessner et al. 2003). In this study, we showed that the elevation of corticosterone in the CMS-treated mice could be prevented by the NAI intervention, and this may be one of possible mechanisms underlying its role in ameliorating CMS-induced depression-like behaviors in mice.

Patients with depression often show signs of inflammation, which was illustrated by the increased concentrations of cytokines such as IL-1, IL-6, and TNF-α in the peripheral blood and cerebrospinal fluid (Leonard 2007; Paudel et al. 2018). It has been reported that administration of IFN-α, used to treat infective diseases, is a significant risk factor in inducing major depressive episode (Miller Andrew H. et al. 2009; Su et al. 2019). Among 20 cytokines examined, two (IL-15 and IL-7) were altered in CMS-treated mice, and more (IL-7, IL-15, IL-21, and TNF-α) were changed by NAI exposure either in control mice or CMS-treated mice. To evaluate possible contribution of cytokines in the ameliorated depression-like behaviors by NAI intervention, we analyzed the alterations of ratios of proinflammatory and anti-inflammatory cytokines, which may provide a whole view concerning the net effects of altered cytokines in modulating brain functions of CMS-treated mice. The ratios of IL-4/IL-2, IL-5/IL-2, and IL-13/IL-2 were elevated in CMS-treated mice. After NAI treatment, two of them (IL-4/IL-2 and IL-13/IL-2) were reduced significantly, each with the level similar to that of control mice respectively. The ratio of IL-5/IL-2 was also reduced although it did not reach the statistical difference. As IL-2 functions as a type I cytokine, the alterations of IL-2-related ratios support the idea that CMS treatment may lead to the shift of Th1 inflammatory pathway with deleterious effects on mood-related brain functions in mice, whereas NAI intervention may prevent or reduce the activation whereby it displays beneficial effect in mood regulation.

### Limitation of this study

Our results indicated that NAI exposure is capable of preventing the onset of depression-like behaviors induced by CMS in mice. The elevation of corticosterone and alteration of some cytokine in the serum of the CMS-treated mice is dismissed after the NAI intervention, but it should be noted that these events are associated with behavioral phenotypes, lacking data for showing any causality between them. This question can be addressed by using cytokine-deficient mice in future studies. It is also unclear how these alterations contribute to brain functions (e.g., the behaviors examined in this study). In addition, it is also interest of investing long-term beneficial effects of NAI exposure for depression-like behaviors.

## Conclusions

Chronic stress is the risk factors in development of depression, and the CMS protocol is widely used in depression animal model, by which we demonstrated beneficial effects of NAI exposure on depression-like behaviors in mice. The present data together with previous findings highly recommend application of NAI in mood-related clinical practice.

## Supplementary Information

Below is the link to the electronic supplementary material.

Supplementary file1 (DOC 4230 kb)

## Data Availability

All data generated or analyzed during this study are included in this published article and its supplementary information files. The datasets used and/or analyzed during the current study are available from the corresponding author on reasonable request.
