# Supplementary material for: Negative air ion exposure ameliorates depression-like behaviors induced by chronic mild stress in mice
Source: Environ Sci Pollut Res Int. 2022 Apr 11;29(41):62626–36. doi: 10.1007/s11356-022-20144-x (PMC9464145; doi:10.1007/s11356-022-20144-x)
Supplement: Supplementary file 1 — Supplementary file1 (DOC 4230 kb) [file 11356_2022_20144_MOESM1_ESM.doc]

**Supplementary Information**

**Article title: Negative air ion exposure ameliorates depression-like behaviors induced by chronic mild stress in mice**

Yun-Qing Hu^1^, Ting-Ting Niu^4^, Jian-ming Xu^5,6^, Li Peng^5,6^, Qing-Hua Sun^7^, Ying Huang^3^, Ji Zhou^2,5,6^, Yu-Qiang Ding^1,3^

^1^State Key Laboratory of Medical Neurobiology and MOE Frontiers Center for Brain Science, Institutes of Brain Science, Fudan University, No.130 Dong’an Road, Shanghai 200032, China

^2^Department of Atmospheric and Oceanic Sciences & Institute of Atmospheric Sciences, Fudan University, No.220 Handan Road, Shanghai 200433, China

^3^Department of Laboratory Animal Science, Fudan University, No.130 Dong’an Road, Shanghai 200032, China

^4^Institute of Occupational Health and Environmental Health, School of Public Health, Lanzhou University, No.199 Donggang West Road, Lanzhou, Gansu 730000, China

^5^Shanghai Typhoon Institute, CMA, No.166 Puxi Road, Shanghai 200030, China

^6^Shanghai Key Laboratory of Meteorology and Health, Shanghai Meteorological Bureau, No.166 Puxi Road,Shanghai 200030, China

^7^School of Public Health, Joint China-US Research Center for Environment and Pulmonary Diseases, Zhejiang Chinese Medical University, No.548 Binwen Road, Hangzhou 310053, China

Correspondence: Ji Zhou, E-mail: zhoujigood@163.com; Yu-Qiang Ding, E-mail: dingyuqiang@vip.163.com.


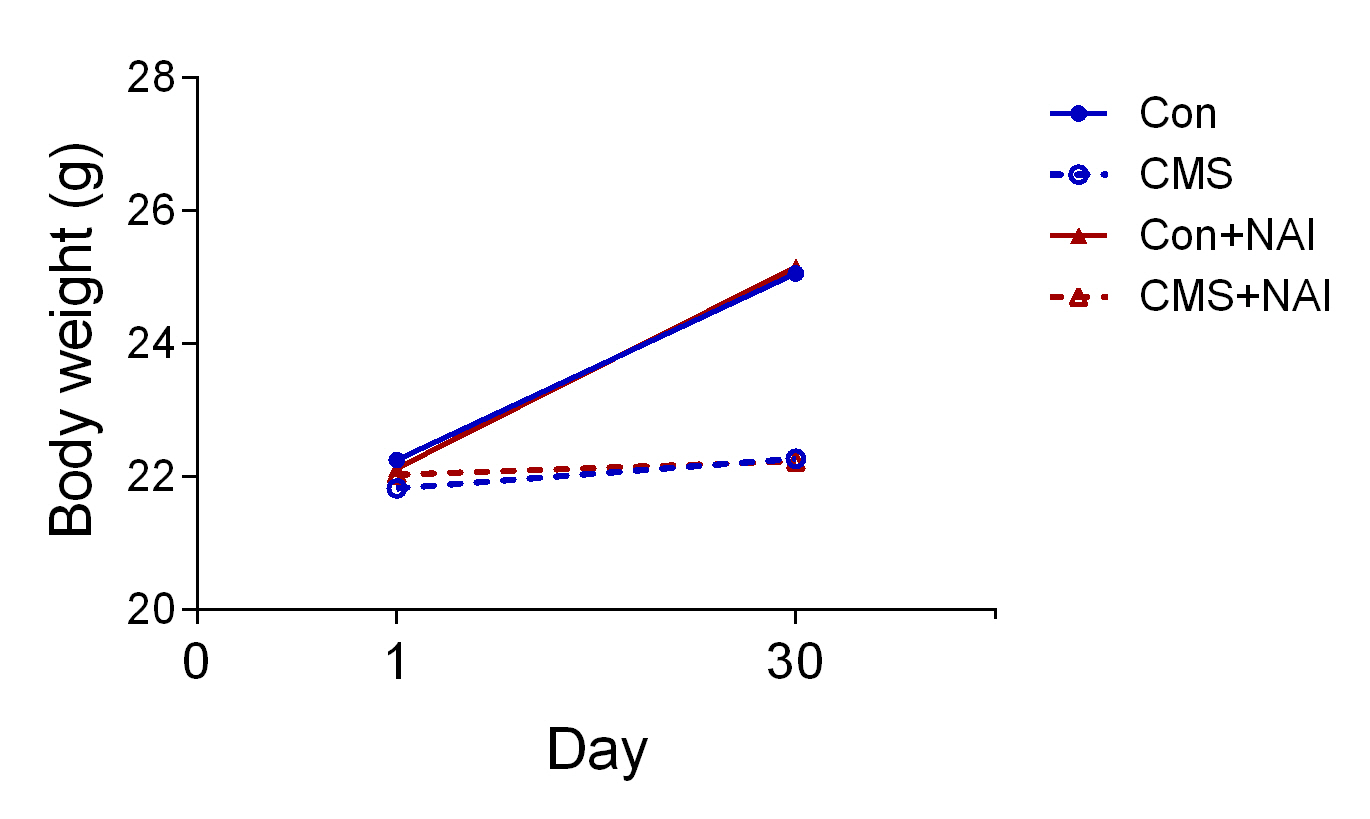


**Fig. S1. The measurement of body weight at the beginning of the study (day 1) and at the end (day 30).**


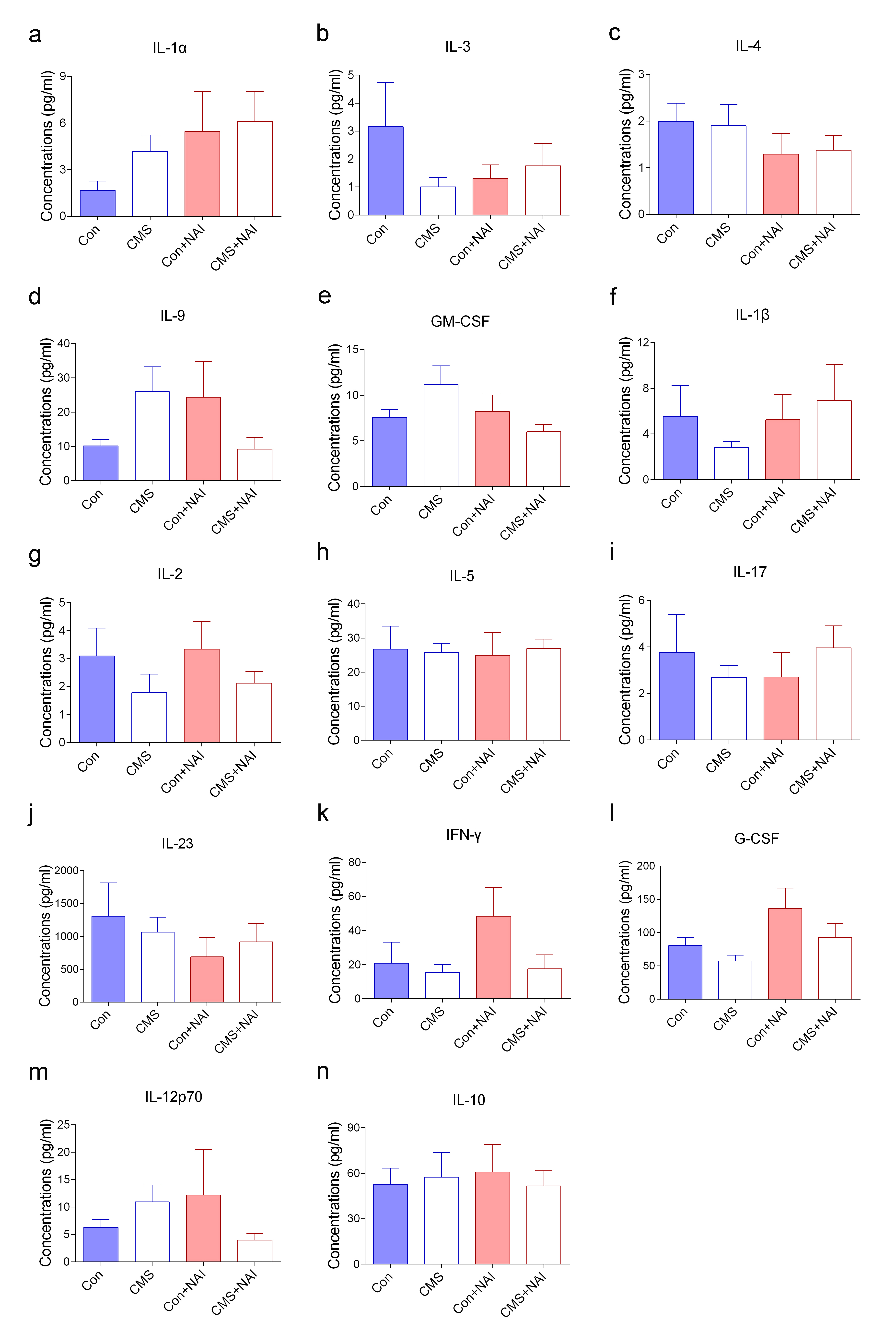


**Fig. S2. Effects of NAI innervation on inflammatory cytokine levels in serum of mice with CMS. (a)** The protein concentration of IL-1α. The one-way ANOVA test with Tamhane’s T2 multiple comparisons test measured the significant differences between these groups {F[3,33] = 1.197, *P* = 0.326 }. **(b)** The protein concentration of IL-3. The one-way ANOVA test with Tamhane’s T2 multiple comparisons test measured the significant differences between these groups {F[3,35] = 1.042, *P* = 0.386 }. **(c)** The protein concentration of IL-4. The one-way ANOVA test with Bonferroni multiple comparisons test measured the significant differences between these groups {F[3,35] = 0.807, *P* = 0.498 }. **(d)** The protein concentration of IL-9. The one-way ANOVA test with Tamhane’s T2 multiple comparisons test measured the significant differences between these groups {F[3,34] = 1.651, *P* = 0.196 }. **(e)** The protein concentration of GM-CSF. The one-way ANOVA test with Tamhane’s T2 multiple comparisons test measured the significant differences between these groups {F[3,35] = 1.908, *P* = 0.146 }. **(f)** The protein concentration of IL-1β. The one-way ANOVA test with Bonferroni multiple comparisons test measured the significant differences between these groups {F[3,33] = 0.498, *P* = 0.686 }. **(g)** The protein concentration of IL-2. The one-way ANOVA test with Bonferroni multiple comparisons test measured the significant differences between these groups {F[3,34] = 0.82, *P* = 0.492 }. **(h)** The protein concentration of IL-5. The one-way ANOVA test with Tamhane’s T2 multiple comparisons test measured the significant differences between these groups {F[3,36] = 0.032, *P* = 0.992 }. **(i)** The protein concentration of IL-17. The one-way ANOVA test with Bonferroni multiple comparisons test measured the significant differences between these groups {F[3,37] = 0.371, *P* = 0.774 }. **(j)** The protein concentration of IL-23. The one-way ANOVA test with Bonferroni multiple comparisons test measured the significant differences between these groups {F[3,37] = 0.592, *P* = 0.624 }. **(k)** The protein concentration of IFN-γ. The one-way ANOVA test with Tamhane’s T2 multiple comparisons test measured the significant differences between these groups {F[3,35] = 1.808, *P* = 0.164 }. **(l)** The protein concentration of G-CSF. The one-way ANOVA test with Tamhane’s T2 multiple comparisons test measured the significant differences between these groups {F[3,34] = 2.477, *P* = 0.078 }. **(m)** The protein concentration of IL-12p70. The one-way ANOVA test with Bonferroni multiple comparisons test measured the significant differences between these groups {F[3,33] = 0.650, *P* = 0.589 }. **(n)** The protein concentration of IL-10. The one-way ANOVA test with Bonferroni multiple comparisons test measured the significant differences between these groups {F[3,35] = 0.095, *P* = 0.962 }. Data are presented as mean ± S.E.M. 9≤n≤11/group.


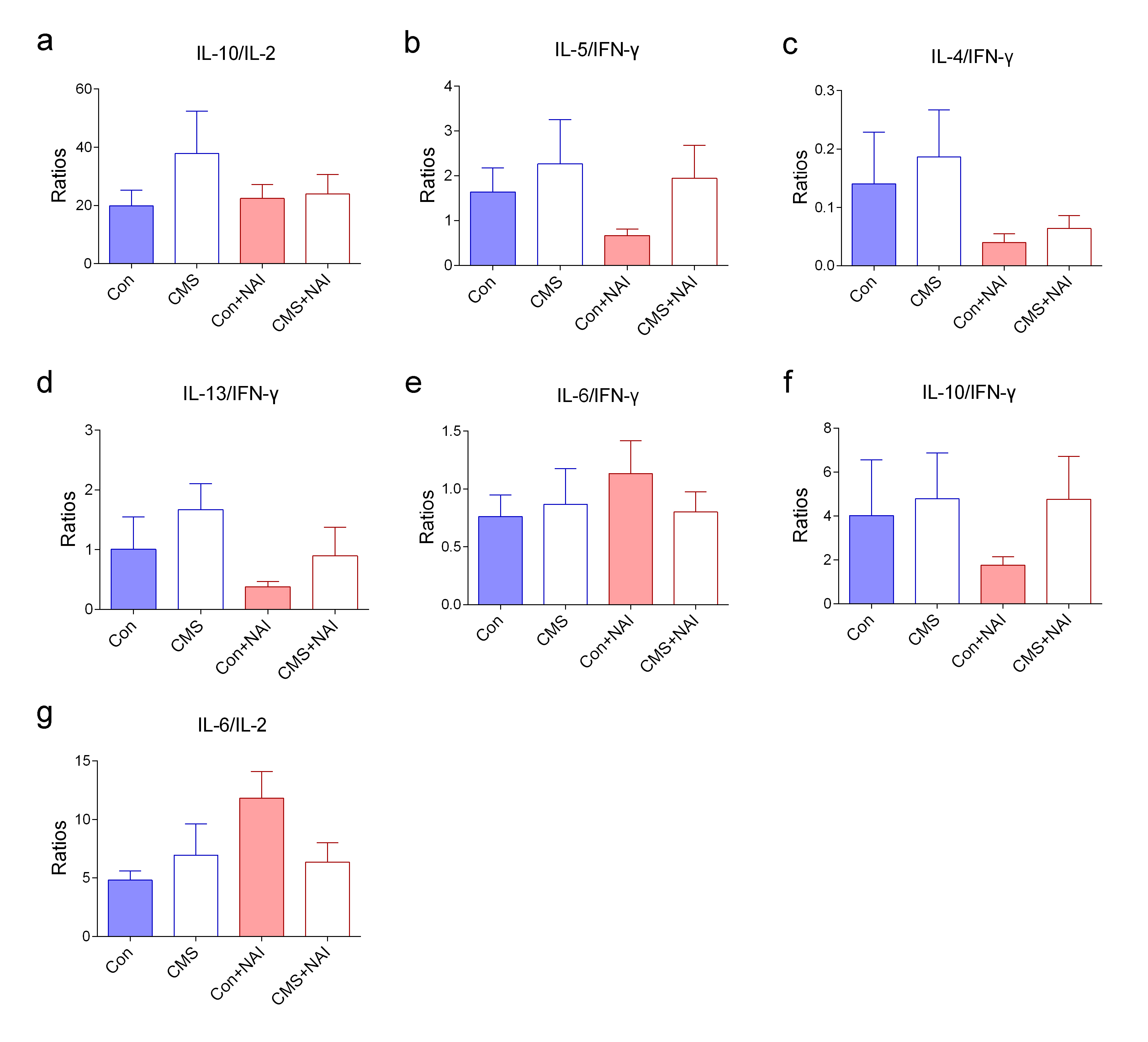


**Fig. S3. Comparison of ratios of IL-10/IL-2, IL-5/IFN-γ, IL-4/IFN-γ, IL-13/IFN-γ, IL-6/IFN-γ, IL-10/IFN-γ and IL-10/IFN-γ in CMS-treated mice with and without NAI exposure. (a)** The ratio of serous concentration of IL-10/IL-2. The one-way ANOVA test with Tamhane’s T2 multiple comparisons test measured the significant differences between these groups {F[3,33] = 0.868, *P* = 0.467 }. **(b)** The ratio of serous concentration of IL-5/IFN-γ. The one-way ANOVA test with Bonferroni multiple comparisons test measured the significant differences between these groups {F[3,24] = 1.072, *P* = 0.380 }. **(c)** The ratio of serous concentration of IL-4/IFN-γ. The one-way ANOVA test with Bonferroni multiple comparisons test measured the significant differences between these groups {F[3,24] = 1.515, *P* = 0.236 }. **(d)** The ratio of serous concentration of IL-13/IFN-γ. The one-way ANOVA test with Tamhane’s T2 multiple comparisons test measured the significant differences between these groups {F[3,25] = 1.899, *P* = 0.156 }. **(e)** The ratio of serous concentration of IL-6/IFN-γ. The one-way ANOVA test with Bonferroni multiple comparisons test measured the significant differences between these groups {F[3,25] = 0.417, *P* = 0.743 }. **(f)** The ratio of serous concentration of IL-10/IFN-γ. The one-way ANOVA test with Bonferroni multiple comparisons test measured the significant differences between these groups {F[3,24] = 0.703, *P* = 0.560 }. **(g)** The ratio of serous concentration of IL-6/IL-2. The one-way ANOVA test with Tamhane’s T2 multiple comparisons test measured the significant differences between these groups {F[3,34] = 2.398, *P* = 0.085 }. Data are presented as mean ± S.E.M. 6≤n≤10/group.
